# MiR-204 enhances mitochondrial apoptosis in doxorubicin-treated prostate cancer cells by targeting SIRT1/p53 pathway

**DOI:** 10.18632/oncotarget.21960

**Published:** 2017-10-23

**Authors:** Yan Shu, Ligang Ren, Bo Xie, Zhen Liang, Jing Chen

**Affiliations:** ^1^ Department of Urology, Tongde Hospital of Zhejiang Province, Hangzhou 310012, China; ^2^ Department of Urology, First Affiliated Hospital, School of Medicine, Zhejiang University, Hangzhou 310003, China

**Keywords:** prostate cancer, doxorubicin, miR-204, SIRT1, p53

## Abstract

Chemotherapy is important for adjuvant treatment of prostate cancer. However, some cancer cells exhibited low sensitivity to chemotherapeutic agents. We are supposed to sensitize these prostate cancer cells to chemotherapeutic agents such as doxorubicin. Previous reports have suggested that microRNAs (miRNAs) regulate chemosensitivity in various cancers. In the present study, we observed that expression level of miR-204 was decreased in prostate cancer cell lines and patients’ tumors. Furthermore, we found that restore of miR-204 dramatically enhanced the cytotoxicity of doxorubicin (DOX) against prostate cancer cell lines C4-2 and LNCaP carrying wild type (WT) p53. Mechanically, miR-204 in prostate cancer cells targets SIRT1 which is a histone deacetylase, and thus decreasing deacetylation of p53. As the results, acetylated p53 induced by DOX upregulates the expression of Noxa and Puma followed by induction of mitochondrial apoptosis. These data demonstrate that restore of miR-204 in prostate cancer cells enhances the mitochondrial apoptosis induced by doxorubicin by targeting the SIRT1/p53 pathway.

## INTRODUCTION

Prostate cancer (PCa) represents as the second most common cancer in men. Furthermore, prostate cancer is the leading cause of cancer-related deaths worldwide [1, 2]. Although surgical therapy is the most effective treatment for early stage PCa patients, chemotherapy is important for postoperative adjuvant treatment and the patients with advanced cancers [3, 4]. However, some PCa cells exhibited low sensitivity to chemotherapeutic agents. We are supposed to take strategies to reduce the drug resistance to achieve effective treatment for PCa patients. Among the chemotherapeutic agents used in PCa, doxorubicin (DOX) is an effective one. DOX acts as a DNA damaging agent by inhibiting the synthesis and repair of DNA, and thus inducing apoptosis of cancer cells [5, 6]. However, some PCa cells exhibit resistance to DOX-induced apoptosis [7]. It is urgent to explore novel strategies to reduce the resistance to apoptosis, and thus improving the efficiency of DOX treatment in PCa.

Silent information regulator 1 (SIRT1) belongs to the sirtuin family and functions as a histone deacetylase [8]. In cancer cells, SIRT1 is associated with cell death/survival and apoptosis by deacetylating important transcriptional factors, including p53, p73, Ku70 and FOXO [9–12]. Recent studies have demonstrated that SIRT1 protects cancer cells from apoptotic signaling when they are treated with DNA-damaging agents [13]. Therefore, SIRT1 is a potential target for improving the efficiency of chemotherapy.

MicroRNAs (miRNAs) are endogenous and single-stranded non-coding RNAs. MiRNAs function as gene suppressors by binding to the 3' untranslated region (3' UTR) of their target mRNAs which contain complementary sites paired with miRNAs. As 60% of human genes are regulated by miRNAs, they are involved in various physiological and disease processes including cancer [14–17]. Recently, studies indicate that dysregulation of miRNAs contributes to low sensitivity to DOX in a number of cancers [18–20]. However, the association between miRNAs and DOX treatment in PCa is still needed to be explored. In the present study, we demonstrate that SIRT1 is overexpressed and targeted by miR-204. The aim of this study is to explore the role of miR-204/SIRT1 pathway in sensitizing PCa cells to DOX treatment *in vitro* and *in vivo*.

## RESULTS

### Expression of miR-204 is decreased in PCa cells

**Figure 1:**
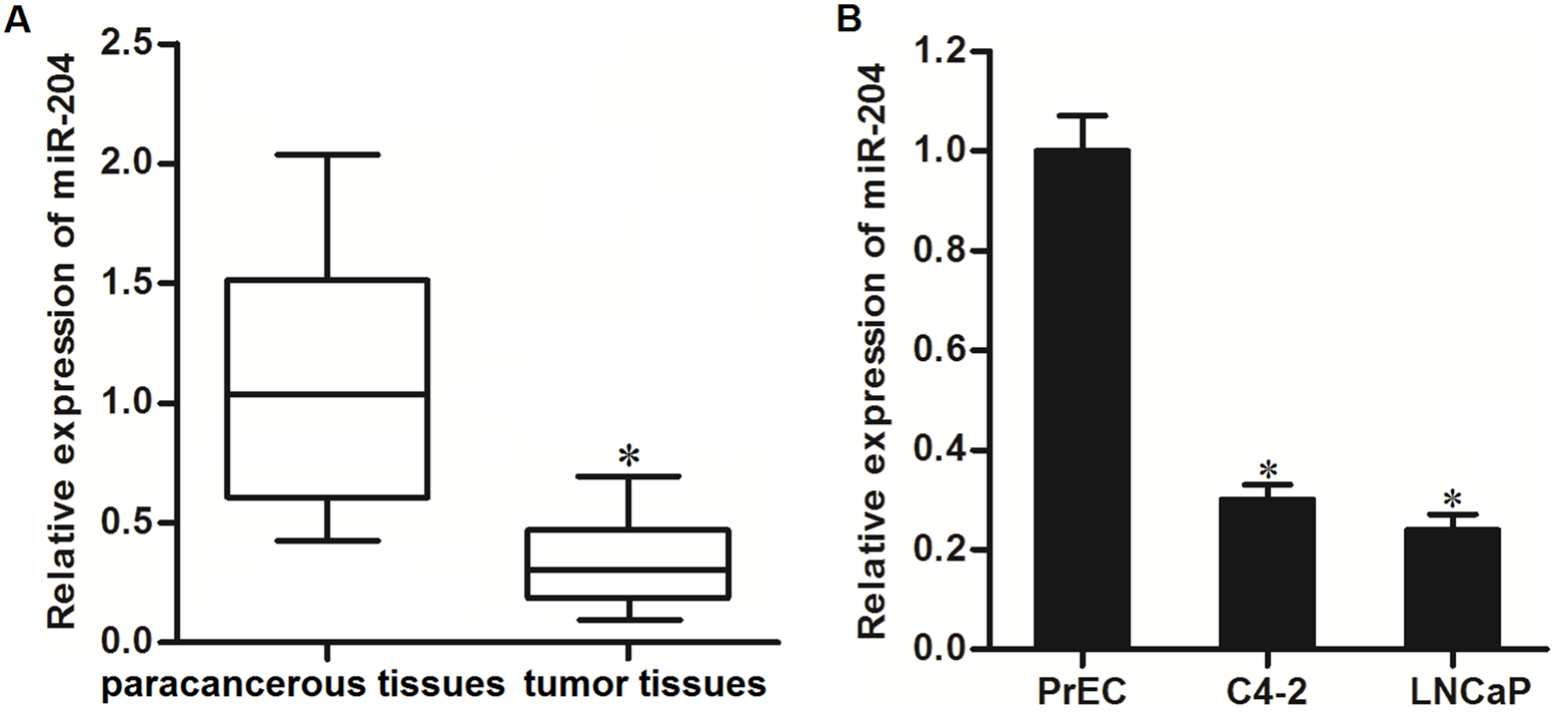
Decrease of miR-204 expression level in PCa **(A)** QRT-PCR analysis was performed to test the expression of miR-204 in PCa patients’ tumor and paracancerous tissues (n=20). ^*^P<0.05 *vs*. paracancerous tissues. **(B)** Expression levels of miR-204 in PrEC, C4-2 and LNCaP cell lines. ^*^P<0.05 *vs*. PrEC cells.

To investigate whether miR-204 is dysregulated in PCa, we tested the endogenous level of miR-204 in PCa patients’ tumor and paracancerous tissues by using qRT-PCR analysis. As shown in Figure 1A, expression of miR-204 was decreased in PCa tissues. In addition, we compared the expression level of miR-204 between normal prostate epithelial cell line PrEC [21] and PCa cell lines C4-2 and LNCaP. We showed significant upregulation of miR-204 in these PCa cell lines compared to the normal one (Figure 1B). These data suggested the potential tumor suppression of miR-204 in PCa.

### Restore of miR-204 sensitizes PCa cells to DOX

**Figure 2:**
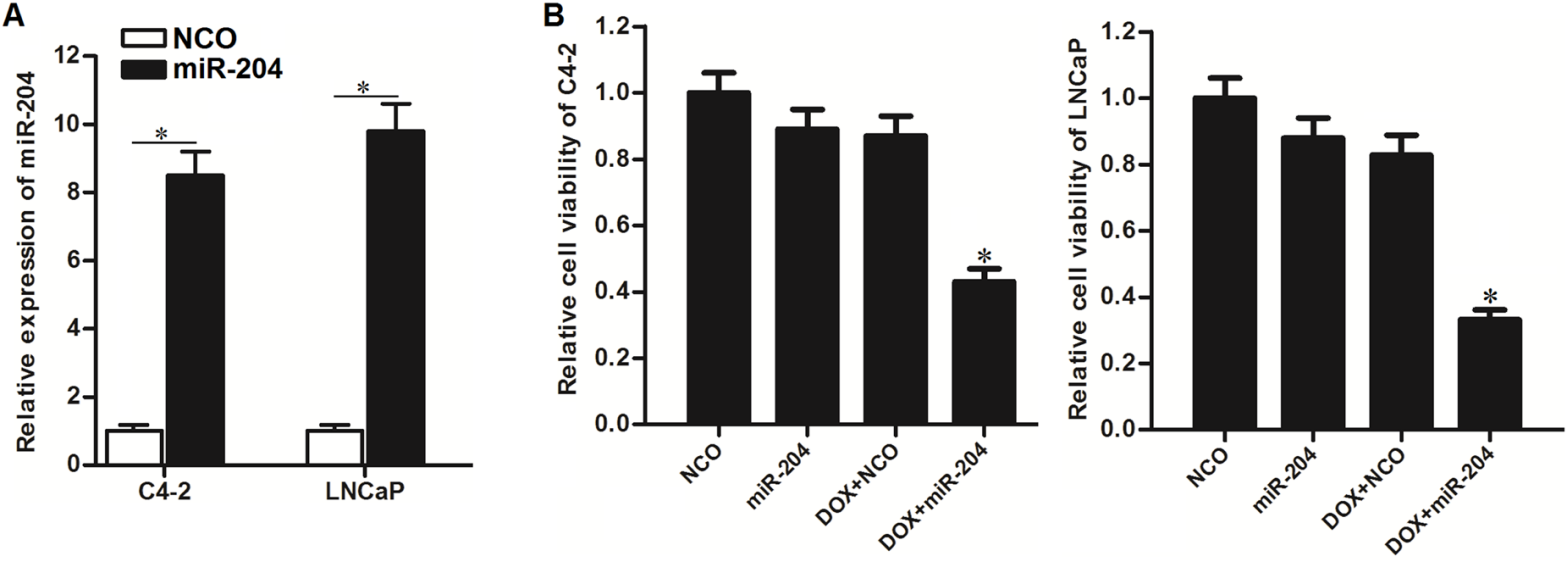
Restore of miR-204 enhanced the cytotoxicity of DOX against PCa cells **(A)** QRT-PCR analysis was performed to test the effect of miR-204 mimic on increasing the cellular miR-204 in C4-2 and LNCaP cells. ^*^P<0.05. **(B)** MTT assays were performed to test the effect of miR-204 and DOX (0.5 μM) on cell viability of C4-2 and LNCaP cells. ^*^P<0.05 *vs*. DOX+NCO group.

As miR-204 was showed to be decreased in PCa, we restored the cellular miR-204 in C4-2 and LNCaP cells by transfecting with miR-204 mimics (Figure 2A) to test its role in DOX treatment. Interestingly, we found that restore of miR-204 dramatically enhanced the cytotoxicity of DOX against C4-2 and LNCaP PCa cells (Figure 2B). We demonstrated that overexpression of miR-204 sensitized PCa cells to DOX treatment *in vitro*.

### MiR-204 targets SIRT1 in PCa

**Figure 3:**
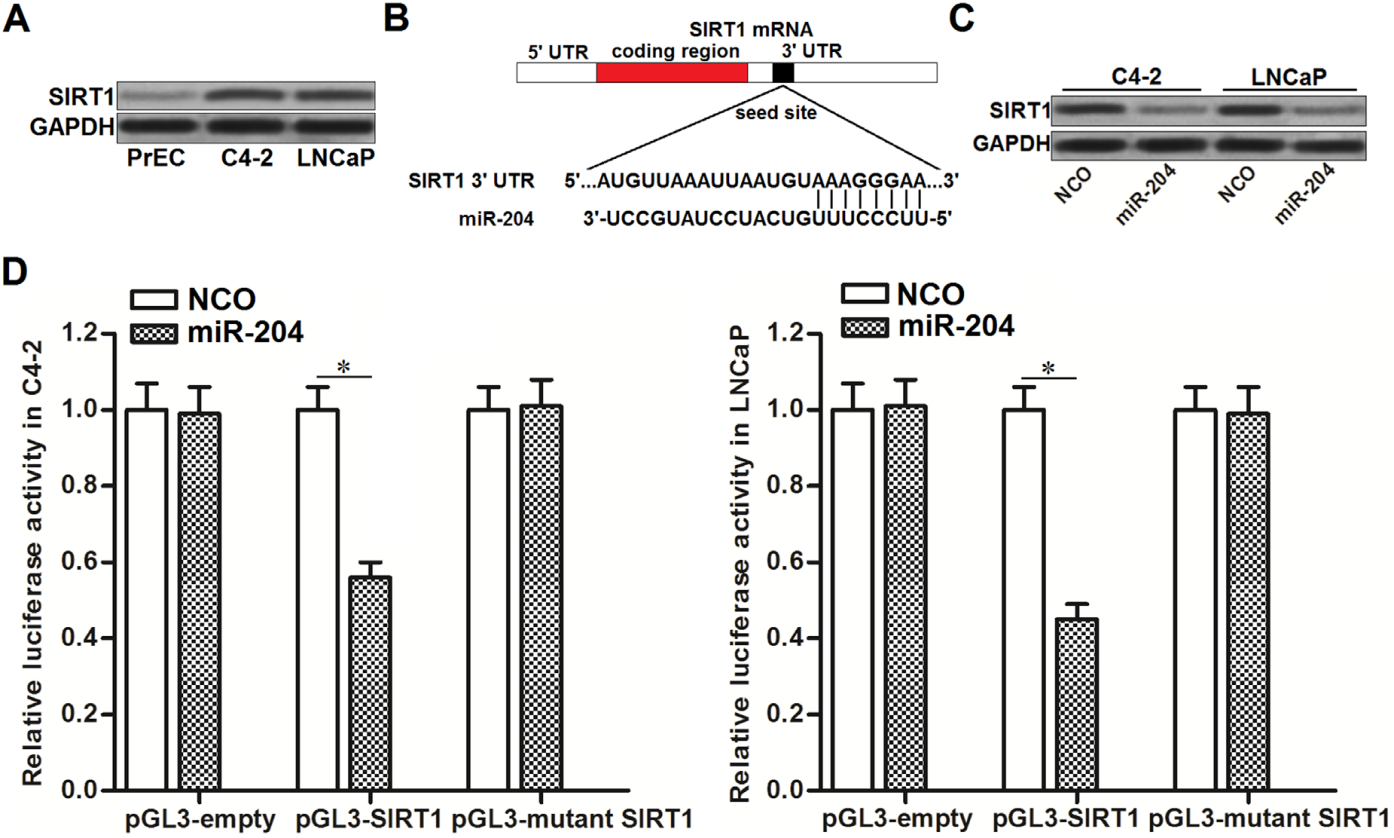
MiR-204 targets SIRT1 in PCa **(A)** Western blot analysis was performed to evaluate the protein level of SIRT1 in PrEC, C4-2 and LNCaP. **(B)** Complementary binding sequence between miR-204 and SIRT1 3’ UTR was predicted by TargetScan database. **(C)** Effect of NCO and miR-204 on changing the protein level of SIRT1 in C4-2 and LNCaP cells. **(D)** Western blot analysis was performed to evaluate the role of miR-29b in changing the expression of SIRT1 in SW480 and OR-SW480 cells. (D) C4-2 and LNCaP cells were co-transfected with SIRT1 3’ UTR reporter plasmid plus miR-204 or NCO. 48 h later, luciferase activities were determined by dual-luciferase reporter assay system. ^*^P<0.05.

As cellular SIRT1 was reported to protect cancer cells from the DNA-damaging agents [13], we detected the expression of SIRT1 in PrEC cells and PCa cell lines C4-2 and LNCaP. We observed obvious upregulation of SIRT1 in PCa cell lines compared to the PrEC (Figure 3A). By contrast, miR-204 was decreased in PCa (Figure 1B). It suggested the negative correlation between miR-204 and SIRT1. To explore the mechanism by which miR-204 sensitized PCa cells to DOX, we searched the TargetScan database (http://www.targetscan.org/) to predict the potential mRNA targets. We found that SIRT1 mRNA contained complementary sequence paired with miR-204 (Figure 3B). Together, these information suggested that SIRT1 is the target of miR-204. To validate the relationship between miR-204 and SIRT1, we tested the protein level of SIRT1 in PCa cells after they were transfected with miR-204. We found that transfection with miR-204 significantly decreased the expression level of SIRT1 in both C4-2 and LNCaP cells (Figure 3C). Furthermore, we showed that overexpression of miR-204 in C4-2 and LNCaP cells decreased the activities of luciferase reporters contained SIRT1 3’ UTR (Figure 3D). Token together, We demonstrated that miR-204 targets SIRT1 in PCa.

### MiR-204 sensitizes PCa cells to DOX through decreasing the expression of SIRT1

**Figure 4:**
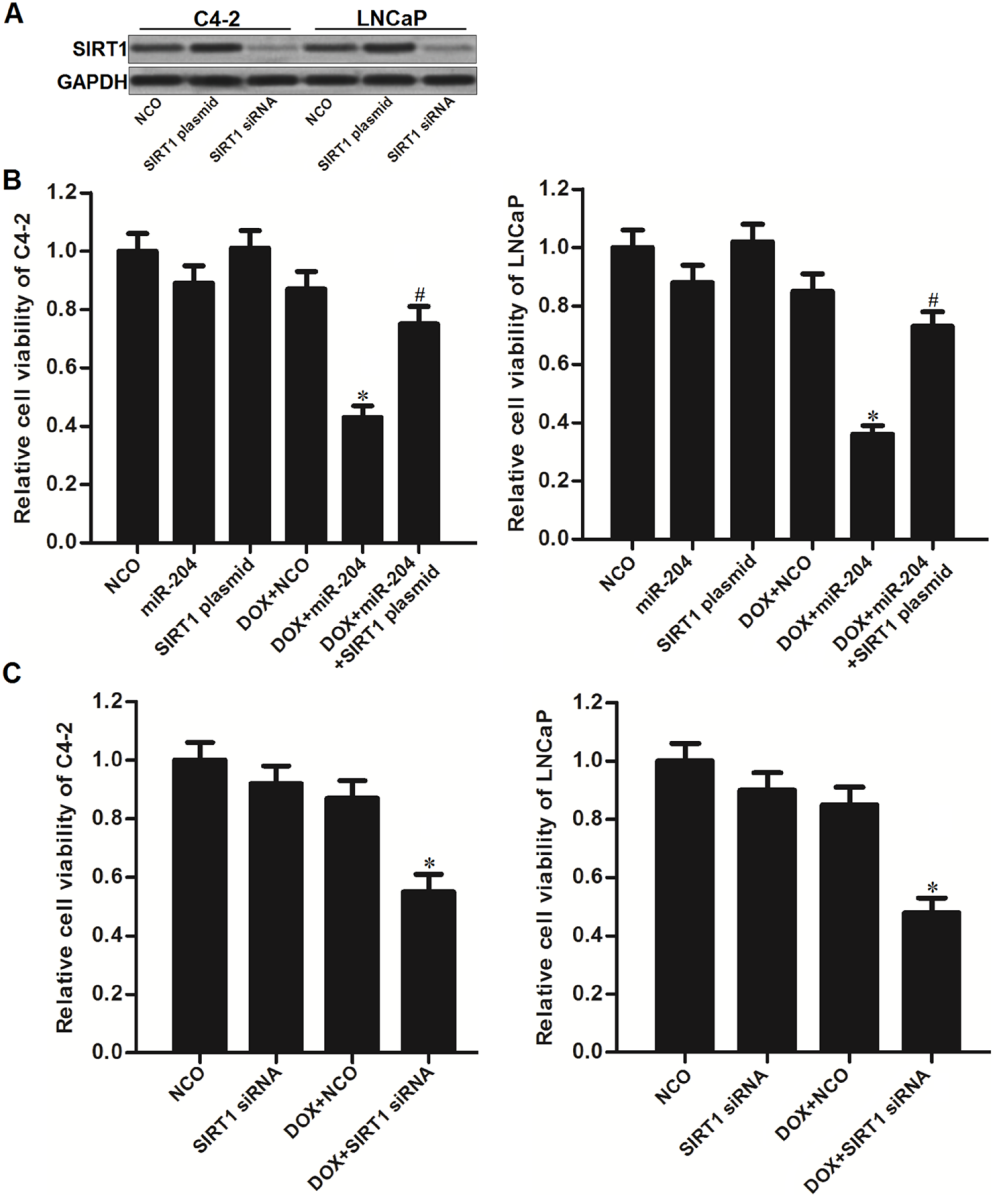
MiR-204 enhanced the cytotoxicity of DOX through decreasing the expression of SIRT1 in PCa **(A)** Effect of SIRT1 plasmid and siRNA on changing the expression level of SIRT1 in C4-2 and LNCaP cells. **(B)** Overexpression of SIRT1 abolished the effect of miR-204 on enhancing the DOX-induced (0.5 μM) cell death in C4-2 and LNCaP cells. ^*^P<0.05 *vs*. DOX + NCO group, ^#^*P*<0.05 *vs*. DOX + miR-204 group. **(C)** Knockdown of SIRT1 dramatically increased the cytotoxicity of DOX (0.5 μM) to C4-2 and LNCaP PCa cells. ^*^P<0.05 *vs*. DOX + NCO group.

To explore the role of SIRT1 in miR-204-promoted cell death, we performed gain- and loss-of-function assays by using SIRT1 plasmid and siRNA (Figure 4A). As shown in Figure 4B, we found that enforced expression of SIRT1 significantly abolished the effect of miR-204 on enhancing the DOX-induced cell death in C4-2 and LNCaP PCa cells (Figure 4B). Moreover, we knockdown the SIRT1 directly by using its specific siRNA, and we found that SIRT1 siRNA dramatically sensitized C4-2 and LNCaP PCa cells to DOX treatment similarly with miR-204 (Figure 4C). These data indicated that miR-204 enhanced the cytotoxicity of DOX through decreasing the expression of SIRT1 in PCa.

### Restore of miR-204 promotes DOX-induced acetylation of p53 in PCa

**Figure 5:**
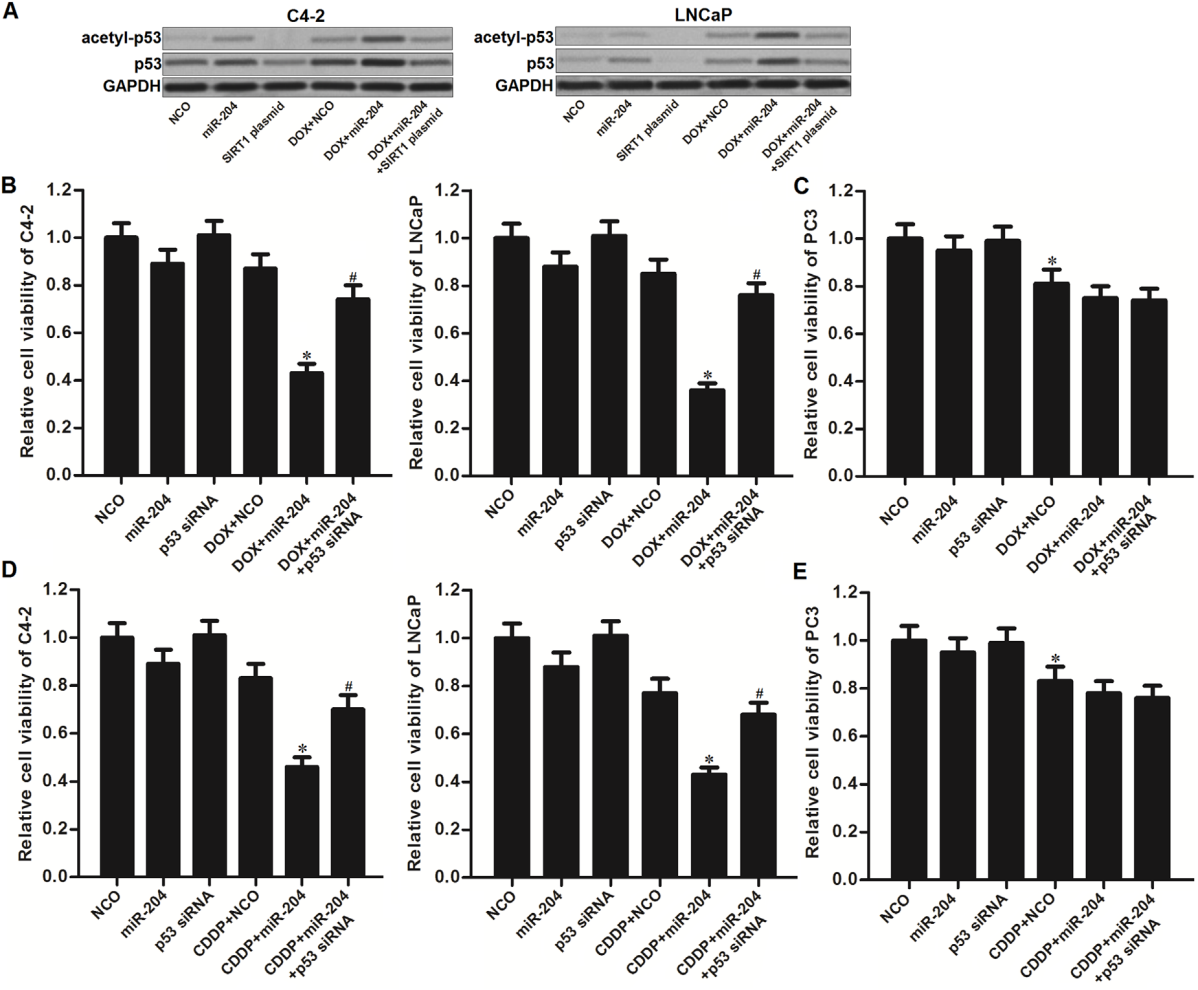
MiR-204 promotes DOX-induced acetylation of p53 in Pca **(A)** Effect of DOX (0.5 μM), miR-204 and SIRT1 plasmid on changing the acetylation of p53 in C4-2 and LNCaP cells. **(B)** Knockdown of p53 abolished the effect of miR-204 on enhancing the DOX-induced (0.5 μM) cell death in C4-2 and LNCaP cells. ^*^P<0.05 *vs*. DOX + NCO group, ^#^*P*<0.05 *vs*. DOX + miR-204 group. **(C)** MiR-204 failed to promote the DOX-induced (0.5 μM) cell death in p53-null PC3 cells. ^*^P<0.05 *vs*. NCO group. **(D)** MiR-204 enhanced the CDDP-induced (2 μM) cell death in C4-2 and LNCaP cells. ^*^P<0.05 *vs*. CDDP + NCO group, ^#^*P*<0.05 *vs*. CDDP + miR-204 group. **(E)** MiR-204 failed to promote the CDDP-induced (2 μM) cell death in p53-null PC3 cells. ^*^P<0.05 *vs*. NCO group.

DNA damage has been reported to induce p53 stabilization and activation by acetylating it [22, 23]. By contrast, SIRT1 induced deacetylation of p53 in cancer cells [9]. As shown in Figure 5A, DOX single treatment induced slight acetylation of p53 in C4-2 and LNCaP cells. On the other hand, combination with miR-204 obviously promoted the DOX to trigger the p53. However, we observed that the acetylated p53 was inhibited by SIRT1 overexpression. These data demonstrated that restore of miR-204 was able to promote DOX-induced acetylation of p53 in p53-WT PCa cells through decreasing the expression level of SIRT1. To test the importance of p53 in miR-204-promted cell death of PCa cells, we knockdown the p53 by using its specific siRNA. Then, we found that the p53 siRNA significantly protected the C4-2 and LNCaP cells carrying wild type (WT) p53 from the cytotoxicity of DOX and miR-204 co-treatment (Figure 5B). In addition, for the PC3 which is the p53-null PCa cell line [24], miR-204 failed to promote DOX-induced cell death (Figure 5C). These data suggested that activation of p53 is essential for the sensitization of miR-204 on DOX-induced cytotoxicity against PCa carrying WT p53. To emphasize the effect of miR-204 on changing sensitivity to other DNA-damaging agent, we co-treated the PCa cells with miR-204 and cisplatin (CDDP). The results showed that miR-204 also can enhance CDDP-induced cytotoxicity to p53-WT C4-2 and LNCaP cells (Figure 5D). However, miR-204 failed to promote CDDP-induced cell death in p53-null PC3 cells (Figure 5E). We demonstrated that overexpression of miR-204 enhanced the anti-tumor effect of DNA-damaging drugs on PCa through the p53 pathway.

### MiR-204 enhances mitochondrial apoptosis in DOX-treated PCa cells through the SIRT1/p53 pathway

**Figure 6:**
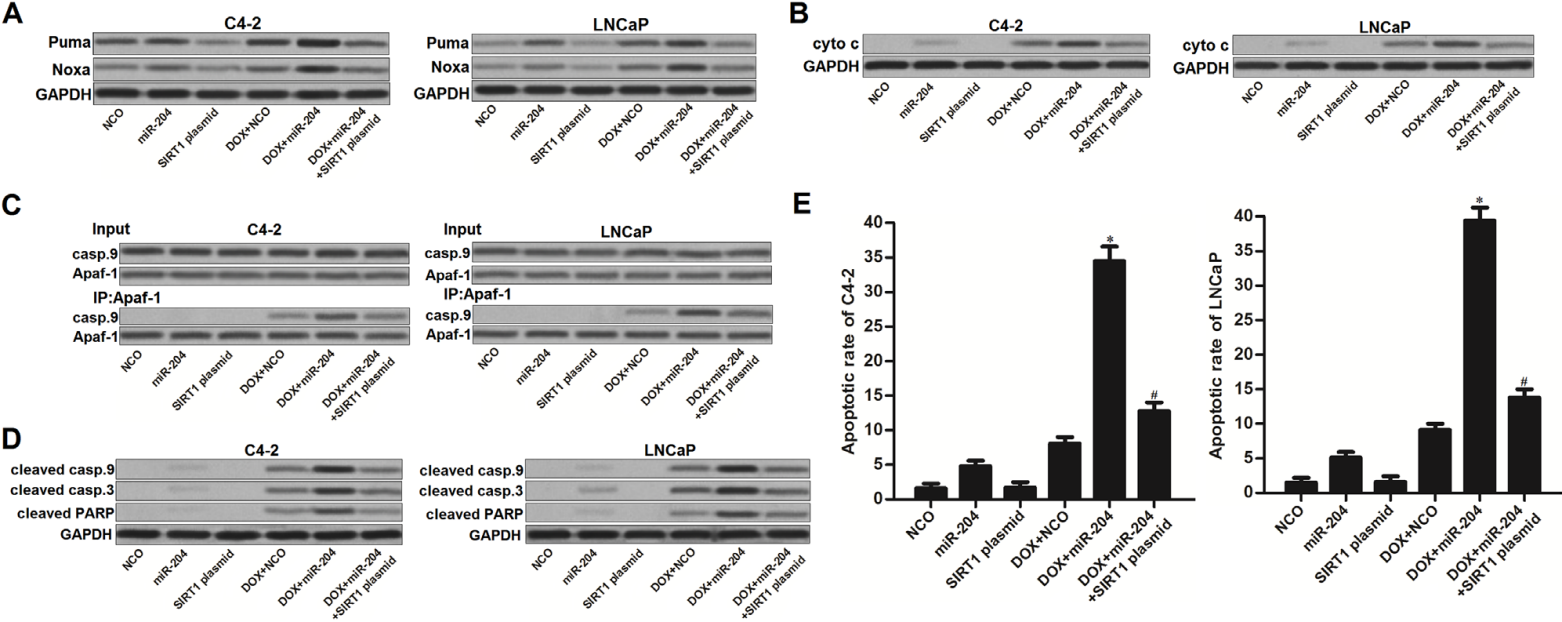
MiR-204 enhances mitochondrial apoptosis in DOX-treated PCa cells **(A)** Effect of DOX (0.5 μM), miR-204 and SIRT1 plasmid on changing the expression levels of Puma and Noxa in C4-2 and LNCaP cells. **(B)** After removal of mitochondria, cyto c in cytoplasm of C4-2 and LNCaP cells was detected by using western blot analysis. **(C)** Interaction with Apaf-1 and casp.9 was tested by co-immunoprecipitation assays in C4-2 and LNCaP cells treated with DOX (0.5 μM), miR-204 and SIRT1 plasmid. **(D)** Effect of DOX (0.5 μM), miR-204 and SIRT1 plasmid on changing the cleavage of casp.9, casp.3 and PARP. **(E)** Apoptotic rate of C4-2 and LNCaP cells was measured after they were treated with DOX (0.5 μM), miR-204 and SIRT1 plasmid. ^*^P<0.05 *vs*. DOX + NCO group, ^#^*P*<0.05 *vs*. DOX + miR-204 group.

As miR-204 targeted SIRT1/p53 pathway in PCa cells, we next investigated the p53-dependent apoptosis in PCa cells which were co-treated with DOX and miR-204. Acetylation of p53 induces the upregulation of downstream Puma and Noxa which induces mitochondrial apoptosis [25, 26]. As shown in Figure 6A, expression level of Puma and Noxa was obviously increased in DOX and miR-204 co-treated C4-2 and LNCaP cells compared to the DOX single treated PCa cells. As the key incidence of mitochondrial apoptosis, we found that combination with DOX and miR-204 induced significant release of cytochrome c (cyto c) from the mitochondria into the cytoplasm of C4-2 and LNCaP cells (Figure 6B). Consistent with the previous research [27], restore of miR-204 in DOX-treated C4-2 and LNCaP cells was found to promote the interaction with Apaf-1 and caspase-9 (casp.9) in the presence of cyto c (Figure 6C). Subsequently, casp.9 was triggered in C4-2 and LNCaP cells. As the downstream of casp.9 activation, casp.3 and its substrate of PARP was obviously cleaved (Figure 6D) accompanying with the occurrence of apoptosis (Figure 6E) in DOX and miR-204 co-treated in C4-2 and LNCaP cells. However, enforced expression of SIRT1 was observed to suppress the mitochondrial pathway of apoptosis in C4-2 and LNCaP PCa cells even they were under the combination treatment with DOX and miR-204. These data demonstrated that miR-204 enhances mitochondrial apoptosis in DOX-treated PCa cells through the SIRT1/p53 pathway.

### Restore of miR-204 enhances anti-tumor effect of DOX on PCa carrying with WT p53 *in vivo*

**Figure 7:**
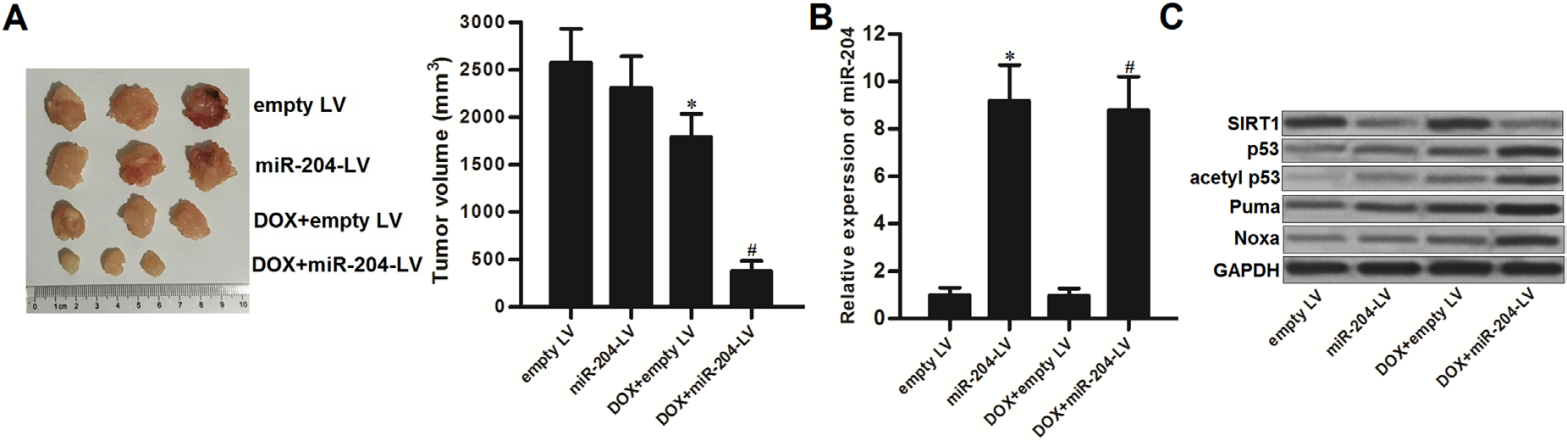
MiR-204 enhances the anti-tumor effect of DOX on LNCaP *in vivo* **(A)** 31 days post inoculation with lentivirus-transduced LNCaP, the final tumor volumes were measured. ^*^P<0.05 *vs*. empty LV group, ^#^*P*<0.05 *vs*. DOX + empty LV group. **(B)** The final tumor tissues cells were purified with collagenase type III. Expression of miR-204 in these tumors was detected by qRT-PCR analysis. ^*^P<0.05 *vs*. empty LV group, ^#^*P*<0.05 *vs*. DOX + empty LV group. **(C)** Protein levels of SIRT1, p53, acetyl p53, Puma and Noxa in the purified tumor tissues cells were detected by western blot analysis.

We established human PCa model by using p53-WT LNCaP cell line on nude mice to test the effect of miR-204 on DOX treatment *in vivo*. We showed that the miR-204-overexpressed PCa tumors were more sensitive to DOX treatment compared to the control PCa tumors (Figure 7A). Next, we purified the tumor tissue cells with collagenase type III to test the expression of miR-204 and its downstream proteins. As we observed significant upregulation of miR-204 in tumor cells transduced with miR-204-lentivirus (miR-204-LV) (Figure 7B), the protein level of SIRT1 was obviously decreased in them (Figure 7C). Furthermore, the downstream of p53 and its substrate of Puma and Noxa was obviously activated in the miR-204-overexpressed tumors under the DOX treatment (Figure 7C). Therefore, we demonstrated that restore of miR-204 was able to enhance the anti-tumor effect of DOX on PCa carrying with WT p53 *in vivo*.

## DISCUSSION

Chemoresistance is still a major obstacle for conservative treatment of various cancers including PCa [28, 29]. Recent studies have demonstrated that expression profile of miRNAs determines the sensitivity of cancer cells to chemotherapeutic drugs. Furthermore, correcting the dysregulation of chemosensitivity-related miRNA has been considered as a promising strategy for cancer chemotherapy [3, 30–31]. Among these miRNAs, miR-204 usually targets oncogenes involved in chemoresistance in multiple cancers. Therefore, miR-204 is reported as a sensitizer to enhance the anti-tumor effect of chemotherapeutic drugs [32–34]. Consistent with these reports, we observed that miR-204 was significantly downregulated in PCa patients’ tumors and PCa cell lines *in vitro*. However, we found that restore of miR-204 increased the sensitivity of the C4-2 and LNCaP PCa cells to DOX treatment *in vitro* and *in vivo*. Therefore, we demonstrated that miR-204 is involved in DOX-based chemotherapy for PCa.

SIRT1 is a protein of histone deacetylase. Usually, it is overexpressed in multiple human cancers, such as colorectal cancer, breast cancer, hepatocellular carcinoma and prostate cancer. Furthermore, increase of SIRT1 has been reported to promote the tumorigenesis, cancer cell growth, metastasis and resistance to chemotherapeutic agents. SIRT1 has been considered as an important target for increasing the chemosensitivity [21, 35–37]. In our study, we identified the SIRT1 as the target of miR-204 in PCa cells. Moreover, we proved that suppression of SIRT1 induced by miR-204 is the mechanism by which miR-204 sensitized C4-2 and LNCaP PCa cells to DOX-induced cytotoxicity.

In miR-204/SIRT1 pathway of sensitization, p53 is a key regulator for DOX-induced apoptosis. p53 is a short-lived protein. Normally, cells maintain a low level of p53 under the unstressed conditions. However, p53 is transiently stabilized and activated mainly through the acetylation modification when the cellular DNA is damaged (for example, DOX treatment in cancer cells) [22, 23, 38]. Subsequently, the acetylated p53 promotes apoptosis of cancer cells by transcriptionally activating several pro-apoptotic genes such as Noxa and Puma in response to DNA-damaging drugs such as DOX [25, 39, 40]. However, cellular p53 can be acetylated by SIRT1 even the cancer cells were under the stress of DOX treatment [38]. Overexpression of SIRT1 inactivates the p53, and thus causing the low response of cancer cells carrying WT p53 to DNA-damaging chemotherapeutic drugs.

In the present study, we found that miR-204-promoted apoptosis induced by DOX was p53-dependent in PCa. Overexpression of miR-204 was able to sensitize the p53-WT PCa cell lines C4-2 and LNCaP, but not the p53-null PC3 cells to DOX treatment. We demonstrated that restore of miR-204 suppressed the expression of SIRT1 and thus stabilizing the DOX-induced acetylation of p53 in p53-WT PCa cells. Subsequently, acetylated p53 increased the expression of Noxa and Puma followed by inducing mitochondrial apoptosis in these p53-WT PCa cells. Our data concluded that miR-204 enhanced p53-dependent apoptosis in DOX-treated PCa cells by targeting SIRT1.

In summary, we prove that restore of miR-204 targets SIRT1 to sensitize p53-WT PCa cells to DOX-based chemotherapy. These findings may represent a novel therapeutic approach for treatment of PCa. However, further studies are required to examine the clinical prospect of miR-204 and its target SIRT1 in PCa chemotherapy.

## MATERIALS AND METHODS

### Tissue samples

Total 20 PCa patients’ tumor and paracancerous tissues were obtained from First Affiliated Hospital, School of Medicine, Zhejiang University from 10/2014 to 8/2016. The use of tumor specimens in the present study were approved by the ethics committee of First Affiliated Hospital, School of Medicine, Zhejiang University. All of the patients had given their informed consent.

### Cell culture

Human PCa cell lines C4-2, LNCaP and PC3 were maintained in RPMI 1640 supplemented with 10% Fetal Bovine Serum (FBS). Human normal prostate epithelial cell line PrEC was cultured in medium recommended by the vendor (American Type Culture Collection, Rockville, MD, USA). All of these cell lines were cultured at 37°C, in a incubator with 5% CO_2_.

### Quantitative real-time polymerase chain reaction (qRT-PCR)

Relative expression of miR-204 was tested by qRT-PCR method. Briefly, total RNA in tissues and cell lines was extracted by TRIzol reagent (Invitrogen, USA) according to the manufacturer’s instructions. Next, One Step PrimeScript miRNA cDNA Synthesis Kit (TaKaRa, China) was used to reversely transcribe the extracted total RNA following the manufacturer’s protocol. Subsequently, the primer (5’-TTCCCTTTGTCATCCTATGCCT-3’) and SYBR Premix Ex Taq (TaKaRa) were used to detect the expression of miR-204 on the Applied Biosystems 7500 Sequence Detection system (Applied Biosystems, USA). Relative expression of miR-204 was normalized to the expression of U6 small nuclear RNA (snRNA).

### Transfection

Mature human miR-204 (5’-UUCCCUUUGUCAUCCUAUGCCU-3’) and negative control oligonucleotides (NCO, 5’-UCUCUUUAUCCUGUCUACCUGC-3’) were purchased from GenePharma Co. Ltd (Shanghai, China). SIRT1 small interfere RNA (siRNA) was purchased from Santa Cruze Biotechnology (USA). For enforced expression of SIRT1 in PCa cells, open reading frame region of human SIRT1 gene was amplified and inserted into the pcDNA3.1 eukaryotic expression vector (Invitrogen, USA). Before transfection, cells were seeded into 6-well plates overnight. 50 pmol/ml miR-204, NCO and SIRT1 siRNA, or 2 μg/ml SIRT1 plasmid was then transfected into the PCa cells by using Lipofectamine 2000 (Invitrogen) according to the manufacturer’s protocol.

### Cell viability assays

The transfected PCa cells were seeded into 96-well plates overnight. Subsequently, cells were treated with 0.5 μM DOX and incubated for 48 h. Cell viability was then evaluated by using 3-(4, 5-dimethylthiazol-2-yl)-2, 5-diphenyltetrazolium bromide (MTT) assay as described previously [41]. The absorbance value of each well was determined at 570 nm by using an ELISA microplate reader (Sunrise Microplate Reader, TECAN, Switzerland). The relative cell viability of each treatment group was normalized to the NCO group.

### Luciferase reporter assays

C4-2 and LNCaP cells were seeded into 48-well plates followed by transfection with the Renilla luciferase pRL-TK plasmid (100 ng/ml, Promega, USA) plus the recombinant Firefly luciferase pGL3 reporters contained 3’ UTR region of human SIRT1 (2 μg/ml, Promega) in combination with miR-204 or NCO by using Lipofectamine 2000. 48 h after transfection, cells were collected and lysed. Luciferase activities were then measured by using the Dual-Luciferase Reporter assay system (Promega) according to the manufacturer’s instructions. Relative Firefly luciferase activities were normalized to Renilla luciferase activities for each tested well.

### Co-immunoprecipitation

Cells were treated with corresponding drugs before incubation with lysis buffer (Cell Signaling Technologies, USA) for 10 min on ice. The resulting supernatants were collected by centrifugation at 12,000 g for 10 min. Protein lysates were then incubated with Apaf-1 antibody (Cell Signaling Technologies, USA) at 4°C overnight at 4°C, followed by incubation with protein A/G plus-Agarose beads for 1 hour at 4°C. Immunoprecipitated pellets were then mixed with the SDS loading buffer and boiled at 95C for 5 mins, followed by western blot analysis.

### Western blot analysis

For detection of cytochrome c (cyto c) in cytoplasm, mitochondria were removed and the cytoplasm was collected by using Mitochondria/Cytosol Fraction Kit (BioVision, USA). For detection of other proteins, whole cell lysates were prepared by using RIPA lysis buffer. Subsequently, protein samples were separated by 10% sodium dodecyl sulfate-polyacrylamide gel electrophoresis (SDS-PAGE) and transferred to a PVDF membrane (Millipore, USA). Proteins on the membrane were then incubated with the primary antibodies and probed by appropriate secondary antibodies. Protein bands were finally detected by using an enhanced chemiluminescent substrate (Thermo Fisher Scientific, Inc, USA).

### Measurement of apoptotic rate of PCa cells

Cells were treated with corresponding drugs before collection for apoptosis detection. Briefly, the collected cells were washed and incubated with Annexin V-FITC and PI (Sigma Aldrich, USA). Subsequently, cell apoptosis was analyzed on flow cytometry (Becton Dickinson, USA). The Annexin V-positive cell population was considered as the apoptotic cells.

### Animal experiments

Before animal experiments, miR-204-overexpressed LNCaP cells (miR-204-LV) were prepared by transfected with recombinant lentivirus carrying miR-204 precusor sequence (Shanghai Genechem Co., Ltd., Shanghai, China). Nude mice (BALB/c, 4∼5 weeks old) were purchased from Shanghai Super-B&K Laboratory Animal Corp., Ltd. (Shanghai, China). For xenograft, 5×10^6^ LNCaP cells (transfected with miR-204-LV or empty LV) were subcutaneously injected into nude mice. DOX (5 mg/kg body weight) was administrated by intraperitoneal injection twice a week. 31 days post-injection, the mice were killed and the tumors were taken out for following experiments. Collagenase type III was used to purify the tumor tissue cells. The animal care and experimental protocols were approved by the Animal Care Committee of Tongde Hospital of Zhejiang Province.

### Statistical analysis

Statistical analysis was performed by using SPSS 15.0. Student’s t-test was used to estimate the statistical differences between two groups. One-way analysis of varianve (ANOVA) was used to determine the differences between three or more groups. Values are expressed as the mean ± standard deviation (SD) and derived from three independent experiments. Differences were considered statistically significant when *P* values <0.05.
